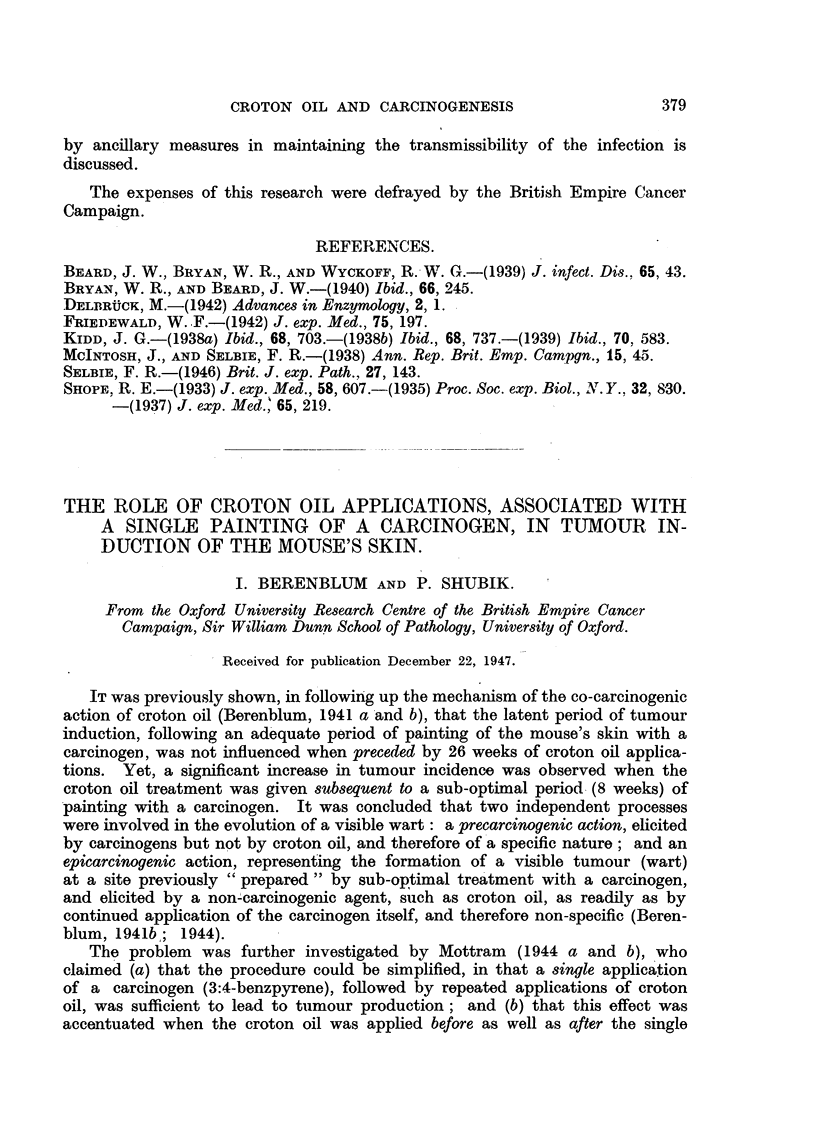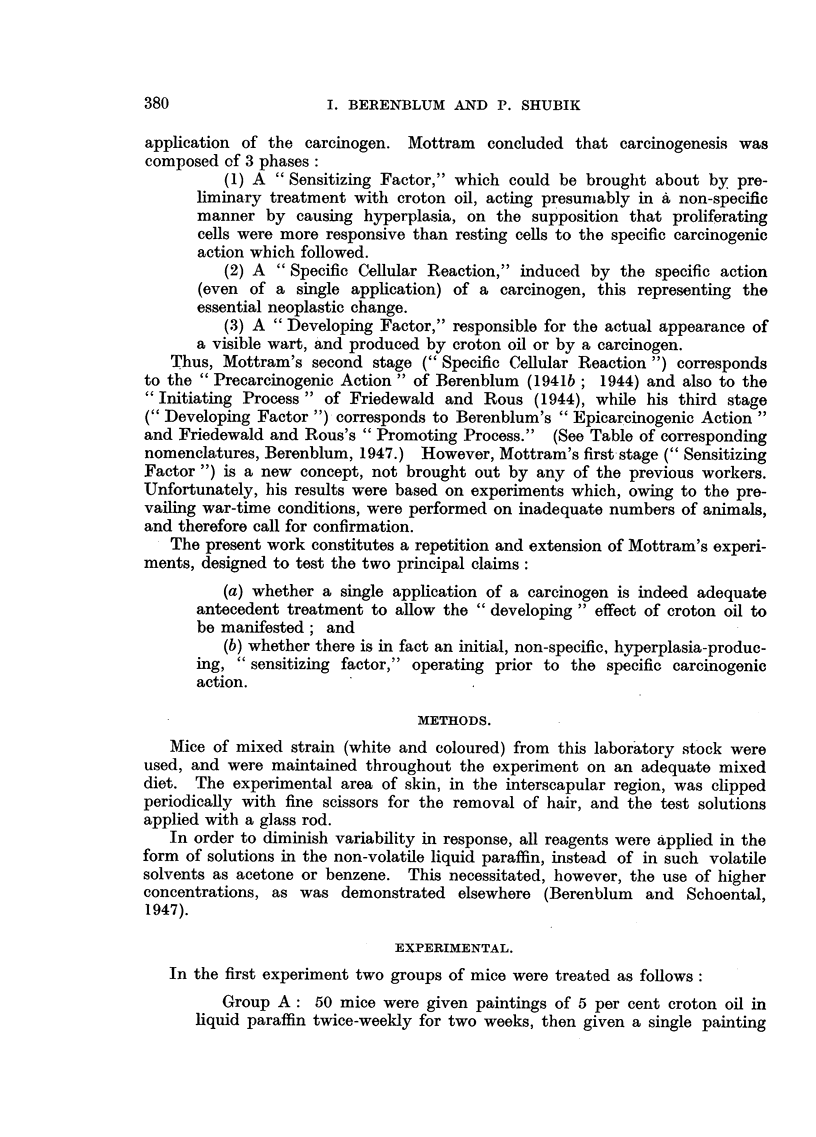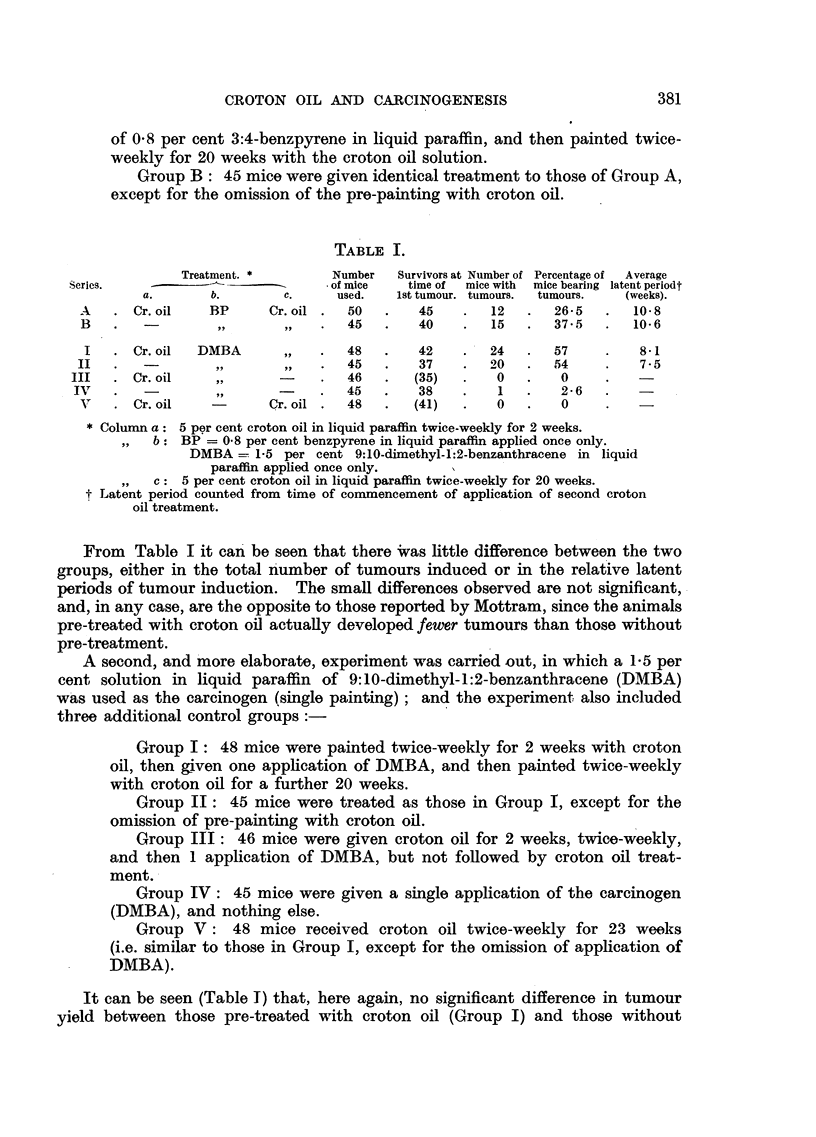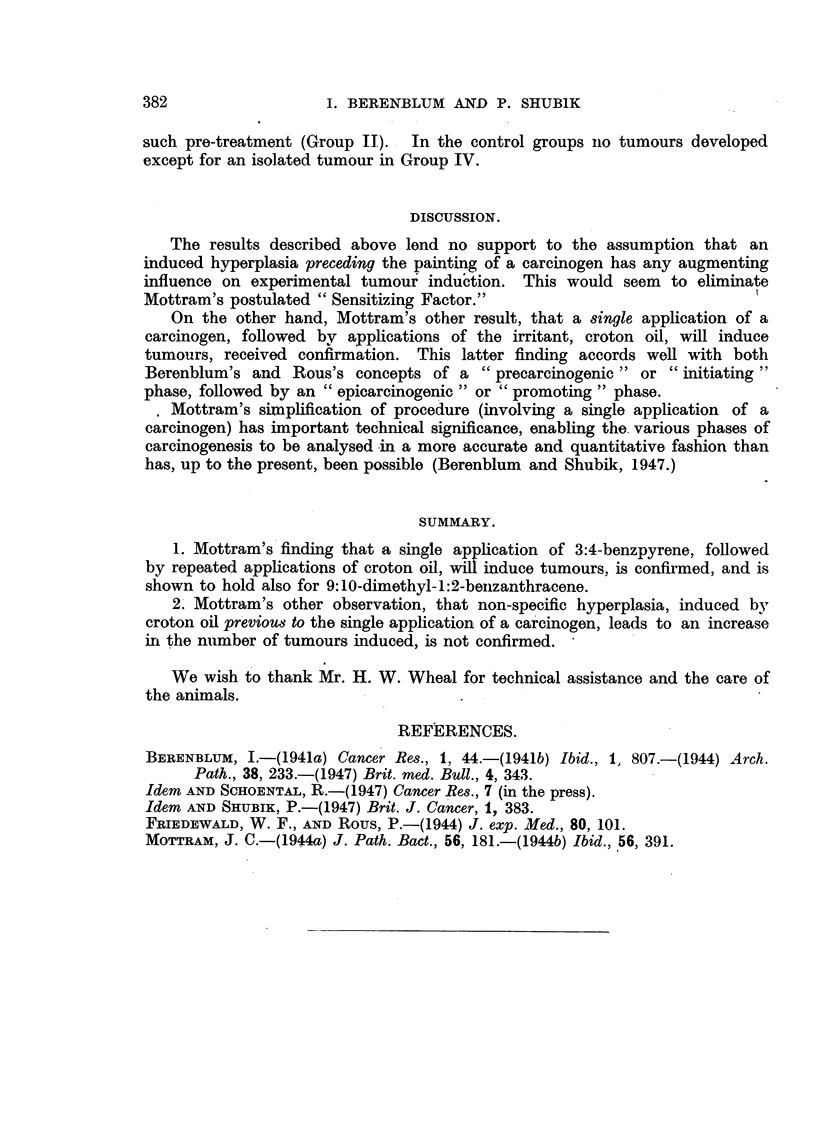# The Role of Croton Oil Applications, Associated with a Single Painting of a Carcinogen, in Tumour Induction of the Mouse's Skin

**DOI:** 10.1038/bjc.1947.35

**Published:** 1947-12

**Authors:** I. Berenblum, P. Shubik


### THE ROLE OF CROTON OIL APPLICATIONS, ASSOCIATED WITH A SINGLE PAINTING OF A CARCINOGEN, IN TUMOUR INDUCTION OF THE MOUSE'S SKIN.

I. BERENBLUM and P. SHUBIK.

*From the Oxford University Research Centre of the British Empire Cancer Campaign, Sir William Dunn School of Pathology, University of Oxford.*

Received for publication December 22, 1947.

It was previously shown, in following up the mechanism of the co-carcinogenic action of croton oil (Berenblum, 1941 *a* and *b*), that the latent period of tumour induction, following an adequate period of painting of the mouse's skin with a carcinogen, was not influenced when *preceded* by 26 weeks of croton oil applications. Yet, a significant increase in tumour incidence was observed when the croton oil treatment was given *subsequent to* a sub-optimal period (8 weeks) of painting with a carcinogen. It was concluded that two independent processes were involved in the evolution of a visible wart: a *precarcinogenic action*, elicited by carcinogens but not by croton oil, and therefore of a specific nature; and an *epicarcinogenic* action, representing the formation of a visible tumour (wart) at a site previously " prepared " by sub-optimal treatment with a carcinogen, and elicited by a non-carcinogenic agent, such as croton oil, as readily as by continued application of the carcinogen itself, and therefore non-specific (Berenblum, 1941*b*; 1944).

The problem was further investigated by Mottram (1944 *a* and *b*), who claimed (*a*) that the procedure could be simplified, in that a *single* application of a carcinogen (3:4-benzpyrene), followed by repeated applications of croton oil, was sufficient to lead to tumour production; and (*b*) that this effect was accentuated when the croton oil was applied *before* as well as *after* the single application of the carcinogen. Mottram concluded that carcinogenesis was composed of 3 phases :

(1) A " Sensitizing Factor," which could be brought about by preliminary treatment with croton oil, acting presumably in a non-specific manner by causing hyperplasia, on the supposition that proliferating cells were more responsive than resting cells to the specific carcinogenic action which followed.

(2) A " Specific Cellular Reaction," induced by the specific action (even of a single application) of a carcinogen, this representing the essential neoplastic change.

(3) A " Developing Factor," responsible for the actual appearance of a visible wart, and produced by croton oil or by a carcinogen.

Thus, Mottram's second stage (" Specific Cellular Reaction ") corresponds to the " Precarcinogenic Action " of Berenblum (1941*b* ; 1944) and also to the " Initiating Process " of Friedewald and Rous (1944), while his third stage (" Developing Factor ") corresponds to Berenblum's " Epicarcinogenic Action " and Friedewald and Rous's " Promoting Process." (See Table of corresponding nomenclatures, Berenblum, 1947.) However, Mottram's first stage (" Sensitizing Factor ") is a new concept, not brought out by any of the previous workers. Unfortunately, his results were based on experiments which, owing to the prevailing war-time conditions, were performed on inadequate numbers of animals, and therefore call for confirmation.

The present work constitutes a repetition and extension of Mottram's experiments, designed to test the two principal claims :

(*a*) whether a single application of a carcinogen is indeed adequate antecedent treatment to allow the " developing " effect of croton oil to be manifested ; and

(*b*) whether there is in fact an initial, non-specific, hyperplasia-producing, " sensitizing factor," operating prior to the specific carcinogenic action.

### METHODS.

Mice of mixed strain (white and coloured) from this laboratory stock were used, and were maintained throughout the experiment on an adequate mixed diet. The experimental area of skin, in the interscapular region, was clipped periodically with fine scissors for the removal of hair, and the test solutions applied with a glass rod.

In order to diminish variability in response, all reagents were applied in the form of solutions in the non-volatile liquid paraffin, instead of in such volatile solvents as acetone or benzene. This necessitated, however, the use of higher concentrations, as was demonstrated elsewhere (Berenblum and Schoental, 1947).

### EXPERIMENTAL.

In the first experiment two groups of mice were treated as follows :

Group A : 50 mice were given paintings of 5 per cent croton oil in liquid paraffin twice-weekly for two weeks, then given a single painting of 0·8 per cent 3:4-benzpyrene in liquid paraffin, and then painted twice-weekly for 20 weeks with the croton oil solution.

Group B : 45 mice were given identical treatment to those of Group A, except for the omission of the pre-painting with croton oil.

TABLE I.

| Series. | Treatment. * a. | b. | c. | Number of mice used. | Survivors at time of 1st tumour. | Number of mice with tumours. | Percentage of mice bearing tumours. | Average latent period† (weeks). |
|---|---|---|---|---|---|---|---|---|
| A | Cr. oil | BP | Cr. oil | 50 | 45 | 12 | 26·5 | 10·8 |
| B | — | ,, | ,, | 45 | 40 | 15 | 37·5 | 10·6 |
| I | Cr. oil | DMBA | ,, | 48 | 42 | 24 | 57 | 8·1 |
| II | — | ,, | ,, | 45 | 37 | 20 | 54 | 7·5 |
| III | Cr. oil | ,, | — | 46 | (35) | 0 | 0 | — |
| IV | — | ,, | — | 45 | 38 | 1 | 2·6 | — |
| V | Cr. oil | — | Cr. oil | 48 | (41) | 0 | 0 | — |

* Column *a* : 5 per cent croton oil in liquid paraffin twice-weekly for 2 weeks.
,, *b* : BP = 0·8 per cent benzpyrene in liquid paraffin applied once only.
DMBA = 1·5 per cent 9:10-dimethyl-1:2-benzanthracene in liquid paraffin applied once only.
,, *c* : 5 per cent croton oil in liquid paraffin twice-weekly for 20 weeks.
† Latent period counted from time of commencement of application of second croton oil treatment.

From Table I it can be seen that there was little difference between the two groups, either in the total number of tumours induced or in the relative latent periods of tumour induction. The small differences observed are not significant, and, in any case, are the opposite to those reported by Mottram, since the animals pre-treated with croton oil actually developed *fewer* tumours than those without pre-treatment.

A second, and more elaborate, experiment was carried out, in which a 1·5 per cent solution in liquid paraffin of 9:10-dimethyl-1:2-benzanthracene (DMBA) was used as the carcinogen (single painting) ; and the experiment also included three additional control groups :—

Group I : 48 mice were painted twice-weekly for 2 weeks with croton oil, then given one application of DMBA, and then painted twice-weekly with croton oil for a further 20 weeks.

Group II : 45 mice were treated as those in Group I, except for the omission of pre-painting with croton oil.

Group III : 46 mice were given croton oil for 2 weeks, twice-weekly, and then 1 application of DMBA, but not followed by croton oil treatment.

Group IV : 45 mice were given a single application of the carcinogen (DMBA), and nothing else.

Group V : 48 mice received croton oil twice-weekly for 23 weeks (i.e. similar to those in Group I, except for the omission of application of DMBA).

It can be seen (Table I) that, here again, no significant difference in tumour yield between those pre-treated with croton oil (Group I) and those without such pre-treatment (Group II). In the control groups no tumours developed except for an isolated tumour in Group IV.

### DISCUSSION.

The results described above lend no support to the assumption that an induced hyperplasia *preceding* the painting of a carcinogen has any augmenting influence on experimental tumour induction. This would seem to eliminate Mottram's postulated " Sensitizing Factor."

On the other hand, Mottram's other result, that a *single* application of a carcinogen, followed by applications of the irritant, croton oil, will induce tumours, received confirmation. This latter finding accords well with both Berenblum's and Rous's concepts of a " precarcinogenic " or " initiating " phase, followed by an " epicarcinogenic " or " promoting " phase.

Mottram's simplification of procedure (involving a single application of a carcinogen) has important technical significance, enabling the various phases of carcinogenesis to be analysed in a more accurate and quantitative fashion than has, up to the present, been possible (Berenblum and Shubik, 1947.)

### SUMMARY.

1. Mottram's finding that a single application of 3:4-benzpyrene, followed by repeated applications of croton oil, will induce tumours, is confirmed, and is shown to hold also for 9:10-dimethyl-1:2-benzanthracene.

2. Mottram's other observation, that non-specific hyperplasia, induced by croton oil *previous to* the single application of a carcinogen, leads to an increase in the number of tumours induced, is not confirmed.

We wish to thank Mr. H. W. Wheal for technical assistance and the care of the animals.

### REFERENCES.

BERENBLUM, I.—(1941*a*) *Cancer Res.*, **1**, 44.—(1941*b*) *Ibid.*, **1**, 807.—(1944) *Arch. Path.*, **38**, 233.—(1947) *Brit. med. Bull.*, **4**, 343.
*Idem* AND SCHOENTAL, R.—(1947) *Cancer Res.*, **7** (in the press).
*Idem* AND SHUBIK, P.—(1947) *Brit. J. Cancer*, **1**, 383.
FRIEDEWALD, W. F., AND ROUS, P.—(1944) *J. exp. Med.*, **80**, 101.
MOTTRAM, J. C.—(1944*a*) *J. Path. Bact.*, **56**, 181.—(1944*b*) *Ibid.*, **56**, 391.